# Allostatic load and mental health during COVID-19: The moderating role of neuroticism

**DOI:** 10.1016/j.bbih.2021.100311

**Published:** 2021-07-30

**Authors:** S. Gallagher, R. Sumner, A.-M. Creaven, P.S. O’Súilleabháin, S. Howard

**Affiliations:** a Department of Psychology, Study of Anxiety Stress and Health, Health Research Institute, University of Limerick, Limerick, Ireland; b Department of Psychology, University of Gloucestershire, Gloucestershire, United Kingdom

**Keywords:** Allostatic load, COVID-19, Inflammation, Mental health, Neuroticism

## Abstract

**Background:**

During the COVID-19 pandemic increased risk of poor mental health has been evident across different cultures and contexts. This study aims to examine whether allostatic load (AL) prior to the pandemic was predictive of poor mental health during the pandemic, and if any associations were moderated by neuroticism.

**Methods:**

Data were extracted from Waves 2 (2011, allostatic load), 3 (2012, neuroticism), and the COVID-19 study (April 2020) of the Understanding Society database in the UK; data were available for 956 participants.

**Results:**

Mental health increased from 2012- to during the pandemic. Neuroticism and AL were positively associated with poorer mental health during COVID-19, such that those who had scored higher on neuroticism and had higher AL prior to the pandemic reported poorer mental health during the pandemic. Neuroticism was also a significant moderator; the effect of AL on mental health during the pandemic was exacerbated in those with high and moderate levels of neuroticism but not lower. Moreover, this was driven by the immune-related indices of AL. This withstood adjustment for age, gender, employment status and prior mental health. These findings are discussed in relation to the pathophysiological mechanisms of mental health.

## 1 Introduction

The negative impact of the COVID-19 pandemic on mental health in a variety of populations has already been noted (Gallagher et al., 2020; Gallagher and Wetherell, 2020) and is now well-established (Vindegaard and Benros, 2020). The theory of ‘allostatic load’ (AL) has been proposed as a model of understanding the physical and mental health consequences of dealing with the prolonged stress, and may well be an important health indicator pursuant to the unique stressors arising from COVID-19 pandemic (Theorell, 2020). This theory posits that repeated or inadequate physiological adaption to stress over time results in *wear and tear* on the body, which mediated through the dysregulation of glucocorticoids via the hypothalamic-pituitary-adrenal (HPA) axis and catecholamine via the sympathetic nervous system (SNS), will result in dysfunction of the cardiovascular, immune, and metabolic systems (McEwen, 1998, 2005), thereby leading to ill-health. AL confers significant physical health decrements for those that experience it; however it may also constitute a precipitative factor for mental health outcomes longitudinally (Juster et al., 2011). Longitudinal assessments of AL also indicate that accumulating stressors can worsen levels of AL, thereby worsening the overall impact to both physical and mental health over time (Piazza et al., 2019). Taken together, this suggests that the accumulation of stress to the point of AL does not end there, and that future stressful experiences, such as the COVID19 pandemic, continue to contribute to AL, and that these impacts also contribute to the manifestation of mental health conditions such as depression.

A recent study found that a psychological index of self-assessed AL (e.g., stress, well-being, psychological distress, abnormal illness and quality of life) was elevated in frontline healthcare workers during the pandemic (Peng et al., 2021). While this study did not examine the underlying physiological indices of AL, other research suggests that there are likely inflammatory biomarkers associated with both COVID-19 infection and mental health (Senra, 2021). In fact, a recent study found that in adult survivors of COVID-19, depression and anxiety symptoms at 1-month follow-up were positively associated with baseline systemic inflammation (Mazza et al., 2020); this is consistent with the notion that inflammation is a key pathophysiological mechanism underlying mood disorders (Miller et al., 2009). Despite this, it is also likely that the effects of AL on mental health during COVID-19 are independent of infection/disease. Evidence for this idea comes from a recent systematic review highlighting that AL was predictive of mental health in both healthy and unhealthy populations (Guidi et al., 2021); as such, it might be that AL prior to the pandemic is predictive of mental health outcomes during COVID-19.

Therefore, the aim of the present study was to establish if AL *prior* to the pandemic was predictive of negative mental health *during* the pandemic. Furthermore, as neuroticism has previously been shown to be associated with AL (Stephan et al., 2016), as well as being predictive of poor mental health (Gale et al., 2016; Nikčević et al., 2021), its role as a potential moderator was examined. Neuroticism is a stable personality trait reflecting the tendency to form negative appraisals of both immediate and long-term stressors (McCrae, 1990), including vulnerability to stress (O’Súilleabháin et al., 2019). According to the diathesis-stress model of psychopathology, an individual possesses some degree of inherent vulnerability (i.e., diathesis) for developing a given disorder (Broerman, 2017), with the onset of a disorder usually triggered by environmental stress (COVID-19). Thus, given that neuroticism is a highly stress-relevant personality trait that moderates physiological reactions to stress (Chida and Hamer, 2008; Hughes et al., 2011) its role in moderating any association between AL and mental health was examined. We predicted that 1) AL and neuroticism would independently predict poorer mental health during COVID-19; and 2) neuroticism would be a moderator of the association between AL prior to the pandemic and mental health during the pandemic.

## 2 Methods

### 2.1 Participants

Our data was obtained from three waves of the Understanding Society study in the UK (Essex., 2010–12); Wave 2 (2011, biomarker study), Wave 3 (2012; personality measures), and COVID-19 first wave (April 2020; first UK lockdown). Participants are a stratified clustered random sample of households representative of the UK general population. The study has ethical approval and each participant gave informed consent prior to participation.

**Fig. 1:**
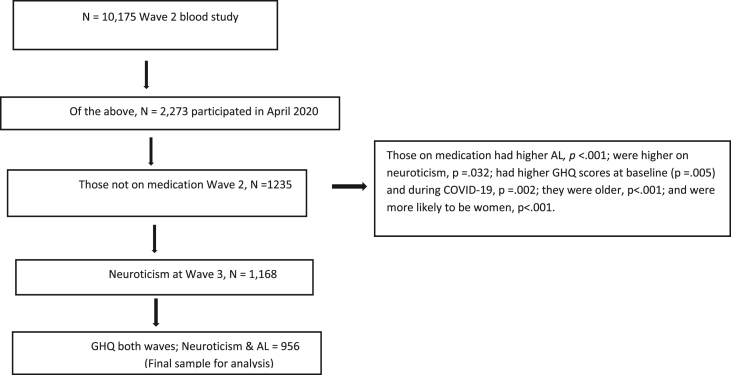
Participant selection flow diagram.

From Wave 2, we extracted AL variables, socio-demographic, and other health-related information that would likely to confound observed relationships (e.g., use of any medication). We dichotomized several of our socio-demographic variables including relationship status (married/partnered vs single/divorced/widowed), education level (college education vs high school or less), and ethnicity (Caucasian/white vs other). Given that use of medication is likely to indicate a health condition, along with influence on the immune and hormonal systems (Rhen and Cidlowski, 2005), participants who reported taking medication (Guidi et al., 2021) (except the contraceptive pill) and/or reported being pregnant were excluded. Participants were only included if they had detectable levels of biomarkers for assessment of AL, and completed the personality assessment at both Wave 2 and the COVID-19 study in April 2020. See Fig. 1 for participant selection and Table 1 for socio-demographics characteristics).

**Table 1:** Participant socio-demographics and correlations among study variables.

	Mean (SD)/%	Ranges	1	2	3	4	5	6	7	8	9	10
**1. Age**	44.91 (13.54)	16–80		.08∗∗	-.25∗∗	.04	-.01	-.04	-.09∗	-.16∗∗	.27∗∗	-.12∗∗
**2. Gender**	47.1% female				.08∗∗	.03	-.04	.01	.06∗	.15∗∗	-.11∗	.17∗∗
**3. Relationship status**	59.2% married					.001	.14∗∗	.003	.04	.08∗	-.01	.12∗∗
**4. Ethnicity**	97.4% White						-.01	-.02	-.06∗	.02	-.03	-.01
**5. Education degree**	45% Degree or higher							.05	.01	.01	.11∗∗	.02
**6. Employment**	75.5% employed								-.03	.001	.002	.06
**7. Baseline GHQ**	1.38 (2.58)	0–12								.45∗∗	-.09∗∗	.33∗∗
**8. Neuroticism**	3.45 (1.34)	1–7									.10∗∗	.23∗
**9. Allostatic load**	4.47 (1.63)	1–10										.03
**10. COVID-19 GHQ**	3.02 (1.64)	0–12	–	–	–	–	–	–	–	–	–	–

Correlations are p ​< ​.05∗; p ​< ​.01∗∗.

### 2.2 Measures

#### 2.2.1 Mental health

Mental health was captured by the 12-item General Health Questionnaire (GHQ) (Goldberg et al., 1997) at Wave 2 and during COVID-19. Items (e.g., unhappy or depressed) are scored as 1 ​= ​not at all; 2 ​= ​no more than usual; 3 ​= ​rather more than usual; 4 ​= ​much more than usual. The scale is a widely used scale, which has intrinsic value as an indicator of psychological distress, particularly in population studies such as this, (Lundin et al., 2016). The continuous scores are used here with higher scores indicate poorer mental health. Internal consistency was high, α ​= ​0.91 for Wave 2 and during COVID-19 α ​= ​0.90.

#### 2.2.2 Neuroticism

This was captured by the 15-item questionnaire on the Five-Factor Model traits (Hahn et al., 2012); neuroticism was assessed with three questions coded on a 7-point scale, where 0 indicated “*does not apply to me at all*”, and 6 indicated “*applies to me perfectly*”. The value range for each trait's total score was 0–18. The internal consistency (Cronbach α) was ​= ​0.69.

#### 2.2.3 Allostatic load

Blood samples for Wave 2 were collected during a single nurse visit, which took place in the participant's home 5 months after wave 2 interviews. In line with the original definition (Seeman et al., 2001), 12 biomarkers representing four biological systems were used in the measurement of AL: the neuroendocrine system (DHEA-s); the immune system (insulin-like growth factor-1 [IGF1], C-reactive protein [CRP], and fibrinogen); the metabolic system (high-density lipoprotein [HDL], low-density lipoprotein [LDL], glycosylated haemoglobin [HbA1C, albumin, waist circumference and body mass index [BMI]); and the cardiovascular system (systolic blood pressure [SBP], diastolic blood pressure [DBP]). These were dichotomized into risk (high vs low) according to quartiles scores or sex-specific risk (e.g., waist circumference), or established criteria (e.g., SBP/DBP ​> ​140/90 and BMI ​> ​25); for some indices (i.e., HDL cholesterol and DHEA-S), the lowest quartile corresponded with the highest risk (T. E. Seeman et al., 2001). As both high and low IFG1 levels have been predictive of morbidity and mortality (Mikkel et al., 2009; Sanders et al., 2018), these two quartiles served as high risk. The risk scores where then coded at 1 ​= ​high risk and 0 ​= ​low risk and summed with higher scores indicating higher degree of risk (Brody et al., 2014; Hawkley et al., 2011; Seeman et al., 2004).

### 2.3 Analytic approach

Prior to hypothesis testing, data were screened for assumptions of fit and normality; all *p*'s for Kolmogorov-Smirnov and Shapiro-tests were >. 05. Following this, descriptives and correlations were used to examine correlations with our outcome variable. This was primarily to identify potential confounds. These were from our baseline (Wave 2) dataset. For our main analyses, we conducted hierarchal linear regressions with confound factors (age, gender, relationship status, and baseline mental health) entered at Step 1, and neuroticism and AL together in Step 2. For our moderation analysis, we used model 1 in Process (Hayes, 2017), to see whether the association between AL interacted with levels of neuroticism.

## 3 Results

### 3.1 Preliminary analyses

As can be seen from Table 1, the sample was middle-aged, predominantly White, mostly in a relationship, employed, and over half the sample did not have a University degree. Older participants had higher AL but better mental health during COVID-19. Allostatic load was higher in men. Women and those who were not married/partnered had poorer mental health during the pandemic. Mental health at baseline (2011) and neuroticism were associated with poorer mental health during COVID-19. These data are for baseline values. However, mental health increased from baseline to follow-up at the pandemic (*t* ​= ​54.55, *p* ​< ​.001). Given the significant associations above with our outcome variable, we controlled for age, gender, relationship status, and prior mental health (measured in wave 2) in our main analyses.

### 3.2 Associations between neuroticism, AL and mental health during COVID-19

In regression analyses, after controlling for confounding in Step 1, both neuroticism, and allostatic load were associated with mental health during COVID-19 9 (See Table 2). Here those who scored higher on neuroticism and who had a higher AL had poorer mental health during the pandemic. The co-variates in Step 1 accounted for 13% (R^2^) of the variance in mental health during COVID-19 and both neuroticism and AL added an additional 1.2% (delta (Δ). We re-ran this regression again looking at both immune and non-immune indices of AL as predictors to see if they offered any unique variance (Wiley et al., 2016). In this instance, both proved to be predictive: immune indices, β ​= ​0.09, 95% CI [0.052, 0.39], *t* ​= ​2.55, *p* ​= ​.01; and non-immune indices, β ​= ​0.08, 95% CI [0.036, 0.29], *t* ​= ​2.50, *p* ​= ​.01. Following this sensitivity analysis, we checked to see if immune or non-immune indices predicted increased depressive symptoms from baseline to follow-up, i.e. change scores. In this analysis, it was the immune-related indices that were predictive β ​= ​.06, 95% CI [0.02, 0.35], *t* ​= ​1.98, *p* ​= ​.04, but not the non-immune, *p* ​= ​.16. Neuroticism was also a predictor in this instance, β ​= ​0.10, 95% CI [0.09, 0.38], *t* ​= ​3.17, *p* ​= ​.02.

**Table 2:** Hierarchical linear regression with demographic, prior mental health, neuroticism and allostatic load predicting mental health during COVID-19.

Variables	β	t	*p*	95%CI Lower	95%CI Upper
Step 1					
Age	-.02	−0.65	.51	−0.19	0.009
Gender	.19	5.89	**.001**	0.69	1.39
Relationship status	.03	0.88	.37	-.19	0.54
GHQ baseline	.29	7.62	**.001**	0.14	0.22
**Step 2**					
Neuroticism	.07	2.07	**.038**	0.008	0.29
Allostatic Load	.09	2.63	**.009**	0.38	0.26

### 3.3 Moderation analyses

After controlling for confounding, the association between AL (total score) and mental health during COVID-19 was moderated by neuroticism, β ​= ​0.08, 95% CI [0.012, 0.152], *t* ​= ​2.32, *p* ​= ​.02. Further, analysis of conditional effects also indicated that the effect of AL on mental health was only significant for those at the medium (3.39), β ​= ​0.11, 95% CI [ 0.016, 0.209], *t* ​= ​2.17, *p* ​= ​.03, and higher (4.71), β ​= ​0.21, 95% CI [0.084, 0.354], *t* ​= ​3.18, *p* ​= ​.001, levels of neuroticism, but not lower levels (2.07), β ​= ​0.001, 95% CI [-0.135, 0.137], *t* ​= ​0.015, *p* ​= ​.98. Moreover, on re-running these analyses again we re-ran this moderation for both immune and non-immune indices for AL and the effects were only evident for immune-related indices of AL, β ​= ​0.13, 95% CI [0.023, 0.233], t ​= ​2.48, *p* ​= ​.01.

## 4 Discussion

This is the first study to demonstrate that AL prior to the pandemic is a risk factor for poor mental health during COVID-19, with the association between AL and mental health moderated by neuroticism. Interestingly, this was only evident for the immune-indices of AL (e.g., IGF1, CRP and fibrinogen). Other studies have also observed similar patterns, but instead of neuroticism moderating the effect of AL on mental health, it was general intelligence that interacted with CRP on mental health (Midouhas et al., 2018). Moreover, these effects withstood adjustment for several important confounders (e.g., age, gender, relationship status, and prior mental health).

Our result for a direct association between AL and future mental health is consistent with the findings of a recent meta-analysis (Guidi et al., 2021). Interestingly in our correlation models (Table 1) AL was not correlated with mental health during the pandemic but after controlling for confounding it was. The effect for neuroticism is similar to that found elsewhere (Nikčević et al., 2021). However, the observed moderation effect of AL on mental health via medium and high neuroticism is consistent with the diathesis stress model of psychopathology (Broerman, 2017) which suggests that individuals possess a vulnerability (i.e., poor coping skills in those high on neuroticism) combined with a biological vulnerability, for dealing with stressors which puts them at risk of poor mental health. Here, we found this moderation was only evident for the immune indices, particularly markers of inflammation, and as such inflammation may be important for the stress-diathesis model. Moreover, our finding for immune-related indices is also in line with current thinking on the role of inflammation in the pathophysiology of mood and other psychiatric disorders (Miller, 2020).

While our study has several strengths (e.g., longitudinal design, population study and controlling for several confounders), there are some limitations. First, there are other unmeasured factors that may be associated with poor mental health during COVID-19 (e.g., loneliness, job loss), and as such, there may be other interactions, as well as other personality traits, that heighten or mitigate the risk of poor mental health. Second, our mental health status was derived through a self-report scale rather than psychiatric interview. Nonetheless, we used a widely used scale, which has intrinsic value as an indicator of mental health (Hahn et al., 2012). Moreover, while it would have been more ideal AL was taken closer to the pandemic, it was taken approximately 9-years earlier, it is similar to other recent testing similar interactions (Midouhas et al., 2018), and in the present study offers a strength in identifying if AL has prognostic power. Further, the power of AL for predicting health outcomes was stronger for 10 years but not at 5 years (Robertson et al., 2017). Further, while AL could remain stable when we encounter unpredictable events in our environments it can increase dramatically and become allostatic overload and damaging for health (B. S. McEwen, 2017). This could have been the case during the current pandemic and perhaps we may see evidence of this emerging in the near future. Our COVID-19 mental health assessment was conducted in the April 2020 wave. Although we anticipate that individuals high in AL might experience a decline in mental health regardless of COVID19, the onset of COVID19 has been established as a highly stressful context during which population mental health has been adversely affected (Mazza et al., 2020). The unique opportunity of this dataset also allowed us to test the diathesis model of psychopathology in which the stressor was present in combination with the psychological and biological vulnerability. Finally, the Understanding Society collects data on an ongoing basis over a 2-year period, there was the possibility of some overlap at the biomarker study and Wave 3 (neuroticism) assessment and this should also be seen as a study limitation. A strength of this design though is that it helped to test the diathesis stress model where exposure to a stressor is needed to test the model (Broerman, 2017) and the COVID-19 dataset allowed us to achieve this by combining all relevant factors.

To conclude, this study confirmed that AL, specifically immune-related indices, is a key factor for predicting future mental health, even when assessed many years previous. In addition, the findings highlight how a stable personality trait such as neuroticism moderates this relationship. Together, the combination of higher neuroticism with higher AL lead to poorer mental health in the COVID-19 pandemic. This highlights that dysregulation across physiological systems, particularly immune-related ones, along with stable tendency to perceive stress, puts individuals at increased risk of poor mental health during times of increased stress.

## Funding

None.

## Declaration of competing interest

The authors have no conflict of interest.
